# ‘It really has made me think’: Exploring how informal STEM learning practitioners developed critical reflective practice for social justice using the *Equity Compass* tool

**DOI:** 10.1080/14681366.2022.2159504

**Published:** 2022-12-20

**Authors:** Louise Archer, Spela Godec, Uma Patel, Emily Dawson, Angela Calabrese Barton

**Affiliations:** a IOE, University College London, London, UK; b Centre for Fusion Learning Innovation and Excellence, Bournemouth University, Bournemouth, UK; c School of Education, University of Michigan, Michigan, USA

**Keywords:** Critical reflective practice, equity, intersectionality, informal STEM learning

## Abstract

Critical reflective practice is a foundation of socially just pedagogy. This paper focuses on the informal STEM (science, technology, engineering, and maths) learning sector, where there is an acute shortage of support for critical reflective practice despite long-standing, entrenched issues of inequity. We analyse how practitioners used a new reflective tool, *the Equity Compass*, co-developed by researchers and practitioners through a five-year partnership. We report on multimodal qualitative data (interviews, ethnographic observations, group discussions, partner site visits, and workshops) from 12 practitioners in four settings in the UK: a community zoo, regional science centre, digital arts centre and an initiative supporting girls and non-binary young people into STEM. We discuss how using *the Equity Compass*: (i) increased and deepened practitioners’ knowledge and understanding of equity issues; (ii) supported personal and institutional critical reflection, helping practitioners move beyond ‘gut instinct’ to interrogate their own positionality, ask new questions, and critically evaluate the effectiveness of attempts at inclusive practice; and (iii) fostered more intentional equitable planning and practice, such as participatory approaches that shared authority with learners and introduced more inclusive forms of representation. We conclude by discussing the challenges, limitations, and implications for supporting critical reflective practice among educators.

## Critical reflective practice and equitable pedagogy

There is a considerable literature discussing the value of reflective practice for educational practitioners and pedagogy. Much of this writing builds on the foundational work of Schön (1991), who noted that the increased complexity within professional settings was associated with a ‘reflective turn’ in practice. Within this reflective turn, the goals of education were broadened beyond the acquisition of content knowledge, to include how teaching and learning can support learners’ informed decision-making, personal growth, and social justice (e.g., Nelson and Sadler 2013).

Despite the popularity of this ‘reflective turn’ in pedagogy, scholars have cautioned that beneath the umbrella of reflective practice lies a considerable conceptual confusion and vagueness in how reflective practice is understood and enacted within education (Kinsella 2010; Ghaye and Ghaye 1998; McLaughlin 1999). In this paper, aligned with our focus on issues of equity and power (within informal STEM learning, or ISL, and the wider STEM education and participation), we focus specifically on *critical* reflective practice and explore how one specific tool (*the Equity Compass*) might help support ISL partitioners towards more equitable practice. As Thompson and Pascal (2012) discuss, critical reflective practice involves questioning one’s own assumptions (i.e., reflexive self-analysis, which they term ‘depth’) and engaging with issues of power and equity (which they term ‘breadth’, or what, in the context of formal education, Larrivee (2000, 294) describes as ‘critical inquiry … the conscious consideration of the moral and ethical implications and consequences of classroom practices on students’). Critical reflective practice extends Schön’s (1991) identification of *reflection-in-action* (reflecting *as* something happens and acting on it immediately) and *reflection-on-action* (reflecting *after* something happens to think about changes are needed going forward) by proposing an additional strand of *reflection-for-action*, including forethought and planning to develop practice (Thompson and Pascal 2012).

Critical reflection thus aims to support learning that ‘transforms problematic frames of reference to make them more inclusive, discriminating, reflective, open, and emotionally able to change’ (Mezirow 2003, 58–59). It involves an ongoing critique of institutions and of repressive forms of authority (van Manen 1977). In this way, as Liu (2015) argues, critical reflective practice helps transform teachers from rigidly following the curriculum to being able ‘analyse and adapt their teaching to particular students in particular social, cultural and political contexts’ (136–137).

Critical reflective practice has been referred to as the ‘highest level’ of reflective practice (Larrivee 2000, 2008; van Manen 1977) and lies at the core of equitable and justice-oriented pedagogical approaches, such as critical pedagogy, culturally relevant pedagogy, and antiracist frameworks (e.g., Arday 2019). Through critical reflective practice, educators seek to understand, identify, reflect on and challenge dominant power relations and practices. As Larrivee (2008) explains:

Teachers who are critically reflective focus their attention both inwardly at their own practice and outwardly at the social conditions in which these practices are situated. They are concerned about issues of equity and social justice that arise in and outside the classroom and seek to connect their practice to democratic ideals […] critically reflective teachers strive to become fully conscious of the range of consequences of their actions.

(343)

Attention has been drawn to the urgency and need to support teachers to understand and address issues of power within their teaching, especially given the dearth of critical discussion and content within many teacher education and training programmes (e.g., Kohli 2019; Ellis, Souto-Manning, and Turvey 2019; Souto-Manning 2019).

Despite the potential promise of critical reflective practice, evidence suggests that some teachers might use reflection merely to confirm their existing beliefs (Fox, Dodman, and Holincheck 2019) and some question whether it is possible, or appropriate, to expect teachers to reflect meaningfully while engaged in the complex act of teaching (e.g., Newman 1996; van Manen 1995). In this paper, we add to the literature though empirical examination of ISL educators’ engagement in critical reflection, focusing specifically on forms of critical reflection that are aimed at transforming social injustice and conducted prior to, or after (but not during) teaching.

Along with the multiple and complex understanding of reflective practice, there is no single commonly agreed model for researching *critical* reflective practice. As Larrivee (2008) notes, there is ‘considerable variability’ in how critical reflective practice is understood and operationalised. Based on our reading of the literature (e.g., Brookfield 1995; Larrivee 2000, 2008; Mezirow 1991, 1997; Thompson and Pascal 2012; Shandomo 2010), we suggest that the general literature on critical reflective practice encompasses the following three main areas, namely that educators:

- Develop *knowledge and understanding of equity/social justice issues*, including understanding relations of power, privilege, oppression, and social reproduction, the significance of language, and how the wider social, political, and cultural contexts shape educational practice and the conditions of teaching and learning.
- Engage in critical *self-analysis of assumptions, values, and practice*, including systematically analysing one’s own beliefs, experiences, and practices of power and privilege, recognising the situatedness of these, and engaging reflexively with feelings of fear, anger, guilt, or shame.
- Engage in *intentionally equitable practice*, which includes *reflection-for-action* – incorporating theory, knowledge, and reflection into the planning and evaluation of one’s practice and future activities, with a particular focus on social justice.

These three areas have informed the analytical framework that we applied in our study to identify and analyse critical reflective practice (see Methods).

Critical reflective practice has been increasingly adopted across various sectors, including education, nursing, social work, management, and organisational theory (e.g., Theobald, Gardner, and Long 2017). Within education, critical reflective practice has been more commonly taken up within the formal sector (e.g., Brookfield 1995; Osterman and Kottkamp 2004; Larrivee 2000), while it has been considerably rarer within the informal sector like ISL which, as discussed next, is an educational field with generally much less staff development, training and support compared to formal learning contexts. Indeed, calls have been made for approaches and tools that can support reflective practice among ISL practitioners (e.g., Moore et al. 2020; Kong 2016; Patrick 2017). Accordingly, in this paper, we seek to add to understandings of how critical reflective practice might be supported within ISL.

As Gottesman’s (2016) useful review explains, the critical turn in education has been enacted through a range of different conceptual and political approaches including (but not limited to) Marxism, feminism, postmodernism, poststructuralism, and critical race theory. In this paper, we do not adopt a singular perspective on critical reflective practice. Rather, we employ an intersectional approach (Crenshaw 1989) that is informed by critical cultural and identity-based scholarship from feminist and critical race scholars, which foregrounds the interplay of gender, racial and class-based forms of power, privilege, and injustice within education (e.g., Hooks 1994; Ladson-Billings 1995).

Reflection is at the heart of emancipatory projects such as feminism and critical race approaches (e.g., Ahmed 2017) and is aimed at supporting educators to understand and challenge unjust power relations through their practice. Accordingly, our understanding of critical reflective practice foregrounds reflection on intersectional structural forms of oppression and the ways in which relations of power and privilege are re/produced (or challenged) through pedagogy. Engaging in critical reflection thus involves actively questioning one’s own pedagogy and seeking to understand and address unjust power relations, values, and forms of representation within and beyond the classroom. In other words, we subscribe to ideas of critical reflection that are aimed at transforming unjust power relations, norms, values and relations of representation and supporting redistribution of social, cultural and symbolic resources, building community through valuing diverse identities, lived experiences and cultural assets. Hence critical reflection approaches pedagogy through the lens of social justice and is a form of social action aimed at challenging hegemony.

## The informal STEM learning context

It is widely recognised that science, technology, engineering, and mathematics (STEM) are elite educational and professional fields in which injustices, such as gender, race/ethnicity and social class, are readily apparent (e.g., Archer et al. 2015). Science education in both the ‘formal’ (e.g., schools, universities) and ‘informal’ (such as science centres, museums, and community science clubs) sectors is marked by structural inequalities, as evidenced by persistent international trends in the profile of post-compulsory and extra-curricular STEM participation. Studies show that both formal and informal STEM learning experiences tend to favour boys and men, as well as those from dominant racial/ethnic communities and those from affluent backgrounds (Bell et al. 2009; Dawson 2019).

Informal STEM learning constitutes a significant area of educational activity. The sector is diverse and comprises a plethora of organisations and initiatives, operating across different scales and budgets. In the UK, mapping conducted by the identified over 600 organisations operating in this space, from small, local grassroots community initiatives, through to professional societies, universities, industry, and the private sector.

Research suggests that ISL settings, but particularly zoos, science centres and science museums, face considerable challenges in creating equitable STEM learning experiences. Studies highlight a range of practices that reproduce and exacerbate injustices through staff and visitor recruitment, representation in collections, interactives, text, way-finding, facilitation, and programming (Cassidy, Lock, and Voss 2016; Das and Lowe 2018; Garibay 2009). Careers in museums and similar organisations are notoriously difficult to enter, with staff typically coming from socially privileged backgrounds (Arts Council England 2021). At the same time, many ISL organisations face considerable funding uncertainty. As a result, ISL settings experience many barriers to developing equitable practice.

In the UK, where this study is located, ISL practitioners face considerable challenges in accessing resources and professional development on how to enact equitable practice. There is a valuable (largely US-based) literature that focuses on issues relating to justice-orientated ISL practice (e.g., Calabrese Barton and Tan 2010; Medin and Bang 2014) and the positive outcomes of this work for young people, communities, and practitioners (e.g., Bevan, Barton, and Garibay 2018). However, it is often difficult to translate this knowledge into practice. Although there are some sector-specific approaches such as the All (2022) project and Museums as Sites for Social Action (MASS Action 2022), not all tools and approaches can be straightforwardly transferred between different types of settings, communities and international contexts (Dawson 2019). This situation is exacerbated by the limited opportunities and resources for professional development and exchanges between research and practice within the field (e.g., Falk et al. 2015).

While calls have been made for the importance of developing reflective practice in the ISL sector (Martin, Tran, and Ash 2019; Patrick 2017; Moore et al. 2020), to date, little work has addressed specifically how ISL practitioners might be supported to engage in *critical* reflection and use this to develop more equitable practice. It is this gap that our paper seeks to fill.

## The equity compass

**Figure 1.**
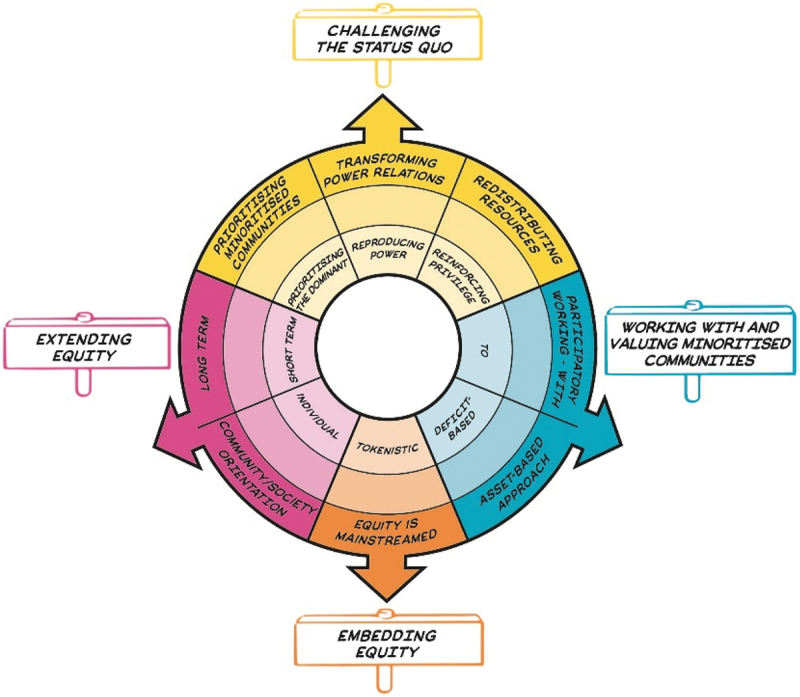
The equity compass.

In this paper, we explore how one tool, *the Equity Compass*, helped support critical reflection among a group of ISL educators and lead to more inclusive practice. *The Equity Compass* is a conceptual heuristic (see Figure 1) that was co-developed by a team of academic researchers and ISL practitioners in the UK as part of a five-year research-practice partnership project (the ‘Youth Equity and STEM’, YESTEM project) in response to ISL practitioners’ need for an accessible tool to support their understanding, thinking about, and planning equitable practice. Rather than providing a script or a list of actions, *the Equity Compass* was designed to support practitioners to engage in critical reflection towards more equitable practice across a range of different ISL settings and activities. In other words, *the Equity Compass* helps ‘orientate’ users to key areas of equitable practice and reflect on and evaluate the extent to which they are working in justice-orientated ways.

*The Equity Compass* is informed by feminist and critical race approaches to social justice (e.g., Hooks 1994; Ladson-Billings 1995), sociological and social justice theories (e.g., Fraser and Honneth 2003), and by equity-focused research in science and STEM education (e.g., Moll et al. 1992). The idea for *the Equity Compass* was prompted by practitioners’ requests for a tool that would practically help them to better understand and articulate what constitutes equitable practice and to be able to map and reflect on their own and their organisations’ journeys. The tool was iterated over time through regular meetings and workshops between researchers, practitioners, and young people, with versions tested and refined through practitioners applying the versions of *the Equity Compass* in their own practice. It was further refined through feedback from formal and informal learning practitioners beyond the project and published in the current form as a practitioner-focused resource in September 2021 (www.yestem.org/tools/the-equity-compass/).

*The Equity Compass* is organised into four overarching areas of equitable practice (Challenging the status quo; Working with and valuing minoritised communities; Embedding equity; and Extending equity). As detailed by Table 1, each area has a set of associated reflective questions, spanning eight dimensions, which aim to support ISL practitioners in critical reflection. The tool supports reflection that is aimed at changing the practice, rather than trying to change learners see also Archer et al. (2017)

**Table 1.** The equity compass dimensions and example reflective questions.

Equity areas	Equity dimensions	Example reflective question
Challenging the status quo	Transforming power relations	To what extent are dominant, unjust relations and conditions being meaningfully transformed?
	Prioritising minoritised communities	Whose interests, needs and values drive the policy and/or practice?
	Redistributing resources	How do experiences support minoritised people – or are efforts mostly directed at more privileged people?
Working with and valuing minoritised communities	Participatory working – with	Is the practice being done ‘to’, ‘for’ or ‘with’ minoritised young people and communities?
	Asset-based approach	How are the interests, knowledge, identities and resources of minoritised young people and communities being recognised and valued?
Embedding equity	Equity is mainstreamed	How central, major, intentional and foregrounded are equity issues in the programme and organisation?
Extending equity	Long term	How is attention being paid to supporting young people’s trajectories and progression over time?
	Community/society orientation	To what extent are the outcomes also collective (e.g., for families, wider communities) and/or the wider field?

This paper explores practitioners’ experiences of how using *the Equity Compass* helped support critical reflection and more equitable practice. Specifically, the paper asks:

- What new knowledge and understanding of equity/social justice issues did practitioners develop through using *the Equity Compass* tool?
- What forms of critical reflection did practitioners develop and practice and what helped/hindered this?
- What forms of intentional equitable practice resulted from practitioners’ critical reflections? What were the limitations?

## Methods

### The research-practice partnership

This paper reports on multimodal qualitative data collected over four years from 2017 to 2021 as part of the ‘Youth Equity and STEM’ (YESTEM) project, funded by the Wellcome Trust, National Science Foundation and the Economic and Social Research Council. The project obtained ethical approval from UCL and adhered to the British Education Research Association’s Ethical Guidelines for Educational Research. The project involved a research-practice partnership (RPP) between a university (UCL) and four partner ISL sites, a community zoo, a science centre, a digital arts centre and an initiative supporting girls and non-binary young people into STEM. The YESTEM RPP was a collaborative, long-term relationship between researchers and practitioners, designed to improve practice (Coburn and Penuel 2016; Penuel and Fishman 2012). Practitioners from each setting were involved in all stages of the partnership, including research design, data collection, analysis, and outputs. Most of the practitioners reported in this paper, together with UK paper authors, were core members of the RPP, taking part in regular in-person and virtual meetings, workshops (setting-specific and cross-setting), reflective discussions, and paired site visits. Funding was provided to cover practitioner time (for about 2–4 days per month) for the entire project.

RPP practitioners were substantially involved in the tool co-development during the first year of the project and were supported by the research team (through the meetings, workshops, calls, and emails) to apply *the Equity Compass* to their own practice in subsequent years. Practitioners used the tool independently with colleagues and, in some cases, with their wider networks.

### Participating practitioners and researchers

12 practitioners took part (Community Zoo = 2; STEM Initiative = 2, Digital Arts Centre = 3; Science Centre = 5), four of whom identified as men and eight as women. Four practitioners identified as racially minoritised and eight identified as White. Practitioners were assigned pseudonyms but we do not provide detailed demographic information for each practitioner participant in order to protect their anonymity. This decision reflects the nature of the research-practice partnership, which included small organisations that are named as project partners in outputs, web materials and so on, and leaves participants particularly vulnerable to identification. Data were collected by Authors 1–5, all of whom identify as women, including two who identified as racially minoritised and four as White.

### Data collection

**Figure 2.**
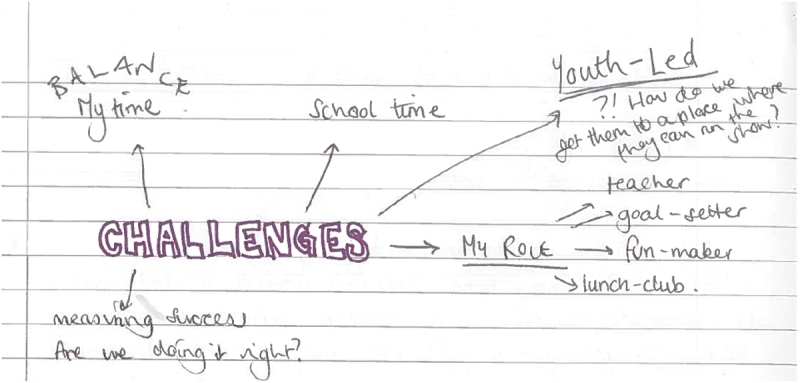
Example of reflection notes.

Qualitative data collection explored practitioners’ perceptions and experiences of the usefulness of the tool, implications for personal and professional practice, and arising challenges. Data included:

- 41 individual semi- and unstructured practitioner interviews (in person and virtual)
- 28 group discussions (in person and virtual)
- 4 all-day site visit meetings and discussions (practitioners visited each other’s settings)
- 54 ethnographically-informed observations of practitioners’ practice within their settings
- 4 project workshops (including practitioners, researchers, young people, and international project partners)
- 4 practitioners-only discussions (meetings recorded by practitioners and shared with researchers)
- 23 practitioners’ written reflections via Google Forms, paper (see Figure 2), and email.

### Data analysis

Data were organised and coded using a theory-led frame derived from the three core areas of critical reflective practice discussed earlier, which we had synthesised from the critical reflective practice literature. The coding resulted in 17 examples coded under *Knowledge and understanding of equity/social justice issues;* 29 examples coded as *Self-reflection on own assumptions values and practice;* and 24 examples coded as *Intentionally equitable action and practice*. These were further delineated by areas and levels of focus (*self, institution and field*, as per Liu 2015; Larrivee 2008; van Manen 1977). Most examples of knowledge/understanding (10/17) and reflection on assumptions (19/29) related to self-focused critical reflection, whereas most examples of intentional action and practice were at the institutional level (14/24).

Data were coded separately by authors 1 and 2, who then checked one another’s codes and discussed discrepancies to produce a final agreed coded set of data. Coded extracts within each of the vertical groupings in Table 2 were analysed thematically to identify the forms of knowledge/understanding, the critical reflection developed, and the resultant changes to action and practice that resulted (within each of the three areas of critical reflective practice, we identified the following themes, namely: language and terminology; privilege, power, and positionality; and different equity dimensions). The analysis and draft of this paper were discussed with practitioners from each of the four organisations, to ensure their voices are suitably represented in the findings.

**Table 2.** Summary of forms of knowledge, reflection and intentional practice developed by practitioners and how the equity compass facilitated these.

Key themes	Forms of new knowledge and understanding of equity/social justice issues	Forms of reflection on own assumptions, values, and practice	Forms of intentionally equitable practice	Role of the Equity Compass tool
Language andterminology	Understanding of social justice language/terminology, concepts and how to articulate these to others	Going beyond ‘gut instinct’ to develop conceptually informed reflection on/for practice	Intentional, equity-informed planning and practice	Introduced new concepts, language, and ideas – and provided a framework to apply to practice
Privilege, power, andpositionality	Understanding of privilege and dominant power relations	Interrogation of their own positionality, values, and behaviours to ask new questions of own and others’ practice	Practitioners advocating for equitable changes in their organisations and/or wider sector	Helped foreground unequal power relations, de-centre dominant perspectives and consider practice through lens of equity.
Different equity dimensions	Understanding of the various dimensions of equity	Recognition of the limitations of previous/initial attempts at equitable practice	Participatory, equitable and representative ISL practice	Outlined key equity dimensions and helped practitioners map and track progress towards deeper equitable practice

## Findings

Table 2 provides an overview of our findings and acts as an orientation point for the subsequent presentation of results and discussion. We begin by discussing findings in relation to each of the three vertical columns, detailing the forms of knowledge, critical reflection, and action that practitioners developed. We then discuss the role of *the Equity Compass* tool in supporting these.

New knowledge and understanding of equity/social justice issues Practitioners came to the partnership with varying prior experience and understanding of equity issues. Some, like Cole and Erin, had been addressing equity issues through their practice for many years. Others, like Madison and Barbara, were newer to the ideas. For instance, during an early project interview, Madison admitted that she was not sure what equity meant (‘I don’t know, sorry’) and described her approach in the work she was doing with young people as: ‘just giving everyone like an equal chance to explore the STEM world that’s out there’. As Kara and Cole both noted, there was a significant dearth of training and support for ISL practitioners to understand and engage with equity issues:

In this institution, there is a massive gap and a huge need for training, and language. They [practitioners] haven’t got the head space for this with all the change happening.

(Kara, group interview)

I didn’t have the training, everything I know I picked up from other people.

(Cole, individual interview).

Irrespective of their starting points, however, all the practitioners felt that using *the Equity Compass* had supported and extended their knowledge of social justice issues, specifically, helping them to: develop new, deeper, and more critical understanding of social justice language/terminology, concepts and how to articulate these to others; understanding of their own positionality, privilege, and dominant power relations; and understanding the various dimensions of equity. While this was felt to be helpful to their own personal development, as discussed further below, it could also be challenging and frustrating when practitioners felt curtailed in their efforts by wider dominant institutional cultures and structures.

### Language and articulation of social justice issues

Across the board, practitioners felt that *the Equity Compass* had provided them with new language and a framework to identify and articulate issues relating to equity and social justice within their practice, which can be understood as their development of ‘equity literacy’ (akin to how critical race reflection can support ‘racial literacy’ among teachers, see Kohli 2019). At the start of the project, many felt that they lacked the appropriate ‘vocabulary’ to be able to effectively reflect on and plan for more socially just practice (e.g., ‘I don’t have the language to explain, no formal training [in equity issues]’, Tessa, site visit discussion). Towards the end of the project, practitioners underlined the value of their new language and understanding. For instance, Barbara felt that before engaging with *the Equity Compass*, ‘I don’t think I’d really thought about equity’ and the tool had ‘definitely given me a better set of language’ (individual interview). For Cole, this new knowledge and understanding gave him confidence and the ability to articulate to others how and why he worked in equitable ways:

Now I can put names to what I’m doing. … . I know the terminology […] I know what I’m talking about now. … . I’m more confident in it […] I can point them to resources.

(Cole, individual interview)

Likewise, Erin explained how using the tool with colleagues ‘has kept us all learning all the time, rather than settling or just being like: “Yes, this is good, what we’re doing”.’ (Individual interview). Specifically, practitioners felt that their understanding was helped by terms and concepts relating to ‘participatory’ approaches, ‘assets-based’ and ‘deficit’ approaches, agency, and by the language around dominant versus minoritised communities’ interests and needs, which we interpret as supporting the practitioners towards new understandings and articulations of the intersectional workings of power within ISL.

### Understanding one’s own positionality

Using the tool helped practitioners to think generally about their own (previously underexamined, taken-for-granted) positionings and how, as Barbara put it, ‘we bring our own orbits with us’ (group interview). For instance, Scott reflected on his own cultural and religious background and positioning and became more aware of how it had shaped his own practice. Cole, similarly, started to think more about the complexity of his own ethnic background and the impact of this on his own values, practice, and his positionality in relation to dominant power relations in the field:

I’m always very aware that I’m normally the only ethnic minority in the room.

(Cole, site visit discussions)

Three of the White practitioners described how, by working with *the Equity Compass*, they had become more aware of their racial positionality and privilege and how this had, in turn, influenced their practice, or as Fook and Askeland (2007, 45) put it ‘factoring ourselves as players into the situations we practice in’. For instance, Tessa described how she now used an awareness of her positionality when reflecting on *the Equity Compass* dimensions, such as when thinking about how to work in participatory ways, recognising imbalances of power and privilege (‘I come as a White middle-class woman asking [minoritised] young people what they want to do’). That is, practitioners from privileged communities began to critically assess and decentre the commonly assumed universality of their own racialised, gendered, and classed identities and experiences.

### Understanding different dimensions of equity

Using *the Equity Compass* over time helped the practitioners to develop new and/or more nuanced understandings of key aspects of equity and social justice, engaging in deeper reflection on particular dimensions. For instance, Tessa reflected during a site visit on what ‘participatory’ practice means and how she might develop it more in her own practice:

It can’t just be a token gesture. You can’t just give someone two options that you think are great and ask them to choose one and go: ‘oh, that’s a great choice!’

(Tessa, site visit discussions)

For two of the practitioners, Tessa and Cole, this understanding led them to reflect anew about how equity issues pertained to their institutions and their wider sectors (science centres and zoos, respectively), or what van Manen (1977, 277), writing about critical reflection, termed ‘a constant critique of dominations’. For instance, Tessa noted the tensions between science centres’ equality and diversity aims, and their standard practice of charging entrance fees. Cole developed a strong critical awareness of the ways in which many zoos (including his own) can struggle to engage meaningfully and justly with issues of race and racism. For instance, discussing the ‘challenging the status quo’ area of *the Equity Compass*, practitioners reflected critically on dominant approaches within their organisation and/or field:

One of our [institutional] aims is ‘diverse participation’. Get more people in the building but all people and not just the current … quite wealthy, middle class, white people. We want our visitors to reflect the diversity of the city, which is an awesome aim, but there are barriers. A big one for me is the entrance fees […] it’s really expensive to get in … It’s a lot of money.

(Tessa, site visit discussions)

Zoos, horrendously, are very white, white, white. And it’s a cultural thing, it’s a class thing. It’s something we struggle with even here. We’re in the most diverse borough in the most diverse city – and we still struggle getting diversity through our gate.

(Cole, site visit discussions)

Cole went on to recall his experience of visiting another zoo, where he had seen racist representations of Black people (‘it was a Black man with a spear and a loin cloth and that’s the only representation’). He linked these experiences to wider intersectional reflections on the zoo sector’s general discomfort with articulating issues of injustice (‘The amount of people that won’t say “gay” or won’t say “Black” as well’) and critiqued the tendency for practitioners to claim not to ‘see’ or recognise injustices (‘“We don’t see disability”, or, “We don’t see colour”, “We don’t see gender”, I’m like, “Well you need to” […] Zoos are a nightmare when it comes to diversity. They are really lagging behind’).

We found that the tool often supported the development and extension of practitioners’ equity-related knowledge and understanding, but particularly within the zoo and the science centre, by supporting practitioners’ awareness of different aspects and dimensions of equity and providing them with a means to understand and articulate what constitutes equitable practice. This new knowledge and understanding of equity/social justice issues then formed a basis for further equity-reflection and practice (Fook and Askeland 2007), which we discuss further below.

### Reflection on own assumptions, values, and practice

In line with the study’s feminist and critical race conceptual framework, we found extensive evidence to indicate that using *the Equity Compass* over time supported practitioners to engage in sustained personal critical reflection that interrogated their own positionality, as well as supporting cultures of collective reflection on these issues within their settings. As detailed in Table 2, we recorded the greatest number of examples from practitioners across all four settings engaging in personal critical reflection with a further four practitioners (one from each of the four settings) also describing collective forms of reflection, interrogating institutional practice. We identified three main forms of critical reflection that practitioners developed and engaged in: going beyond ‘gut instinct’ to develop conceptually informed reflection on/for practice; interrogation of their own positionality and values and asking new questions of own and others’ practice; and recognition of the limitations of previous/initial attempts at equitable practice.

#### Going beyond ‘gut instinct’

Over time, using *the Equity Compass* helped practitioners to reflect more regularly and critically on their practice, moving away from what some termed an approach based on ‘intuition’ or ‘gut instinct’, towards a more structured and deliberate form of reflective practice. Acting on ‘unexamined common sense’, Brookfield (1995, 4) argued, ‘is a notoriously unreliable guide to action’. Barbara explained:

So it has really made me think … in a more structured way about things as well, rather than operating under this feels right or this feels wrong. I think that’s a big shift. […] I think it’s deepened my practice … it’s really focused much more on how we work.

(Barbara, group interview)

Practitioners from each setting also conveyed how *the Equity Compass* had been useful as a tool for supporting collective critical reflection with colleagues. For instance, Erin explained the value of this within the digital arts centre:

I think our main thing [change] would be stopping and reflecting on what we’re actually doing. … Just like really reflecting, sitting, being like: ‘What are we doing, why are we doing it?’

(Erin, individual interview).

Cole, similarly, conveyed the value of collective critical reflective practice in the zoo:

Just checking in on ourselves, I think that’s the biggest thing that changed. I think we talk about it a lot.

(Cole, group interview)

#### Interrogating positionality and asking new questions of own and others’ practice

Building from their self-reflections and enhanced understandings, practitioners started to reflect critically on their previous practice and ask new questions. For instance, Madison described her realisation that some of the resources that she had produced previously ‘were mainly about white women’ and used this insight to inform her work going forward.

Many of the practitioners described how using the tool led them to adopt a ‘questioning approach’ that challenged previous assumptions and practices in their settings, particularly those that reproduced unjust power relations (Thompson and Pascal 2012). For example, both Scott and Tessa talked about how they had started to ask more ‘disruptive’ questions of their own and their institution’s practice, e.g., challenging ‘where [leaders] decide to put resources’ (Scott, site visit discussions), and demanding that decisions are made based on equity and social justice, rather than profit. As Barbara recognised, it was not enough for her to focus only on her own practice – social justice requires organisational change:

I think for it to be disruptive or transformative, it’s got to change our practice as a science centre in some way. It’s got to have an impact on us.

(Barbara, practitioners-only discussion)

Barbara went on to think through what this would mean for specific initiatives and areas of work, asking ‘what would that need to be to make it disruptive? What would we actually have to do?’ Like others, she found that using *the Equity Compass* helped identify concrete areas for change and improvement, both personally and institutionally, including re-thinking previous programme outcomes and goals. For instance, Barbara changed the stated outcomes for her practice as being to support young people’s ‘empowerment’ rather than to increase their STEM skills, knowledge, or aspirations. Bobbi took this point further, suggesting that *the Equity Compass* provided not only a useful articulation of the importance and nature of equity issues, but constituted a tangible tool that she could use to hold ISL leaders and funders more accountable for considering equity in their practice:

… the compass has been a good way to show people, ‘Hey, you do have to go back and think, what is it that makes people not feel they belong or what is it that makes people not promote women?’ … And how do you look forward now for equitable practice or ways that you can do things that allow for, plan for difference?

(Bobbi, group interview)

#### Recognising the limitations of previous practice

Cole and Tessa found that using *the Equity Compass* helped them to identify shortcomings in their previous attempts at participatory practice, such as offering participants choices from a limited set of predetermined options rather than using meaningful co-construction (‘We often tell people, here’s [option] A and here’s [option] B, which one do you want? That’s where we often fall down’, Cole, site visit discussions).

Now, after using the compass up against not only stuff that we do, but stuff that we’ve done … in hindsight, there were areas where it was lacking or where it was missing.

(Cole, individual interview)

Tessa’s reflections also led her to fundamentally interrogate her own role, organisational values and indeed the wider STEM participation agenda that drove much of her own practice:

How are we defining value? What is my role here? … do [participants] really need more STEM in their life? If I’m asking them what they want and they want to do all other things, why should I insist on science? […] It’s thinking, what do we think is valuable, what does that person think is valuable? … You’re so focused on the thing you’re meant to be doing … [that] we don’t stop and go, ‘I need to hand over a bit of agency, let people decide what they actually want to do’.

(Tessa, site visit discussions)

Practitioners in each of the settings described how they used the tool within collective reflection to identify areas of practice that would benefit from change and improvement and areas of complacency. For instance, Bobbi discussed how she felt that her organisation had not been sufficiently clear that trans young people were included in their girls’ programmes. Tessa reflected ‘we are thinking about it [equity], but I don’t know if we always get it right’. As Cole added:

I think one of the biggest changes has been the sort of recognising areas where we might be too comfortable … too complacent, you know?

(Cole, group interview)

We interpret these examples as reflecting Larrivee’s (2000) call for deep examination of personal values and beliefs and, we would add, positionality. As we discuss further below, this process was not always comfortable for practitioners. Indeed, early in the project, Tessa described how she often felt like she was ‘struggling’ to develop her practice in more equitable ways and worried that she was ‘failing’, as exemplified by a following early reflection on the challenge of enacting more participatory practice:

At the moment, I don’t have options to give them [the young people] to let them go ‘hey, this is what I want’ … That’s where I’m struggling. […] That is also something that’s new to me personally, so I haven’t been facilitating that as well as I’d like to.

(Tessa, individual interview).

While at the time of the above interview, Tessa did not feel that she had managed to translate her critical reflections into practice (a process that the compass later helped her with), as Schön discusses, importantly these reflections did open up new possibilities for more equitable practice: ‘When a practitioner becomes aware of his *[sic]* frames, he also becomes aware of the possibility of alternative ways of framing the reality of his *[sic]* practice’ (Schön 1991, 310).

### Intentionally equitable practice

Above, we considered how the practitioners used *the Equity Compass* in ways that developed their knowledge/understanding of equity and supported their personal and collective critical reflection. We now consider how knowledge and reflection were translated into intentional equitable practice through: intentional, equity-informed planning and practice; advocating for equitable changes in their organisations and/or wider sector; and participatory, equitable and representative ISL practice.

### Intentional, equity-informed planning and practice

Practitioners in all settings felt that the tool supported more intentional planning. As Erin explained, at both an individual and collective level, practitioners now asked themselves and each other questions that foregrounded equity and social justice (‘What can we do better, what else can we do? Why are we doing it?’)

I think, that … probably one of the biggest things that’s changed [is] … we all think about it [equity] more. It’s much more intentional, we’re not just doing it because we’re doing it. We’re doing it because that’s the right thing to do and [we ask] ‘what can we do differently’? ‘What are we thinking about and what are we not thinking about?’

(Erin, individual interview)

As Kevin reflected in a group interview, the tool helped colleagues to plan intentionally in a more collective way (‘I think one of the nice things is it allows other people to get involved’). Bobbi and Madison also discussed how they had developed a new, more participatory goal for their STEM club programme (‘we want to get to the point where […] most of the time it’s run by the girls’, Bobbi, practitioners-only discussion). This more intentional approach to practice and planning was further exemplified by Cole in relation to both his own practice (‘A lot of the things that I did before this … I did just because it felt right and nothing more. Whereas now, there are words that I can use to describe it and I know why I’m doing it’, Cole, individual interview) and his organisations’ practice:

[Before] it was sort of a complacent [approach]. But now using something like this [the Equity Compass] helps to make sure that that doesn’t happen. So, I think that’s the biggest positive that’s changed.

(Cole, group interview)

Practitioners felt that the visual and practical nature of the tool, with the articulation of eight specific dimensions of equitable practice, helped them and their colleagues to plan in a more targeted way, map progress and ‘see what’s missing’. As Barbara said, ‘It’s great to say “we measured it on the Equity Compass and look, it fell short”, so we re-addressed it’; individual interview). Cole similarly agreed, ‘I think the compass just brings you back to actually, just make sure each of these points are covered’ (group interview):

To have that visualisation [to see] I could do a little bit better there and I think I’m doing very well there or very poorly there. That, for the way that I do things, that works really, really well for me.

(Cole, individual interview).

#### Advocating for equitable changes in the organisation and the wider sector

Practitioners in three of the settings felt that the Compass had been useful in enabling them to advocate more effectively for change in their organisations in line with social justice principles. As Barbara explained:

When you’re not able to articulate these very specific terms, you’re less able to make a case at any level within your organisation. This [the Equity Compass] … helps you articulate it better.

(Barbara, individual interview).

Cole also discussed how *the Equity Compass* ‘has had a direct effect’, helping him in ‘convincing other people to do similar projects’, obtain funding for equity-focused work and convince other organisational leaders ‘that these practices are not only beneficial to young people but they’re beneficial for your organisation too’ (Cole individual virtual interview). Bobbi similarly explained how she had used the tool to advocate more broadly for change within her sector in relation to addressing gender and ethnic underrepresentation in STEM and challenge some industry-led initiatives aimed at diversifying entry into STEM careers:

I also sometimes will use the compass in talks that I’m doing with companies, to be like, ‘Here’s how this might translate for you and your company, what are the things you’re going to do to ensure that people are part of the way that you’re making decisions or that you’re able to kind of empower people more than just kind of giving lip service to particular things?’

(Bobbi, group interview).

#### Participatory, equitable and representative informal STEM learning practice

We identified various examples of practitioners’ equitable practice deepening, specifically through more participatory and representative practice. For example, in the science centre, Tessa and Barbara organised some initial sessions with colleagues to explore the dimension of participatory practice and engage in collective critical reflection:

So … we’re focusing on – what does participation mean to us? How do we start to understand it? How are we applying equitable practice? Are we applying it without thinking about it?

(Tessa, site visit discussions).

After these initial sessions, practitioners started trying out new ways of sharing authority with the young people in their programmes, e.g., inviting participants to have more say in the focus and delivery of sessions (‘we learnt a lot by doing that’, Tessa, individual interview). The practitioners welcomed and valued the feedback from participants and used it to inform further reflection and intentional planning. Barbara explained:

What we try to do when [participants] communicate something with us is that on reflection we like to go back and say: ‘You said this. We listened to you. So, this is how we will redirect our project, so we can take on board your suggestions’.

(Barbara, individual interview).

By the end of the project, and as a result of their critical reflection using a range of tools (including but not limited to the Equity Compass) the design of the science centre’s youth programmes had evolved considerably, from previously largely facilitator-devised and led sessions, to more participatory models that were responsive to and informed by youth participants.

The zoo developed several new programmes to expand their work with minoritised communities, informed by Cole’s systematic application of the Equity Compass across all their existing programmes to consider how they might be made more equitable. As Cole explained:

The amount of programmes for vulnerable people has expanded … , and I think that’s partly down to again, mine and Kevin’s new knowledge [and] confidence in what we’re doing. […] We had seven or eight programmes that we’ve run. We’ve gone through them all with the Equity Compass.

(Cole, individual interview)

In addition to engaging with minoritised communities, Cole also set up a new youth board, to support more participatory ways of working with young people and to give young people more authority and voice within the workings of the zoo. He and the young people on the youth board used the Compass to continue to critically reflect on and through the lens of equity to intentionally plan their practice.

### Factors that helped or hindered use of the Equity Compass to support critical reflective practice

We identified three main factors that facilitated practitioners’ use of the tool to support critical reflection and equitable practice. First, practitioners felt that the co-constructed nature of the tool gave it a closeness to practice that significantly enhanced its effectiveness. As Madison explained, it was effective because it had been developed by

… taking that theory and taking all of that work and discussions and then actually putting it into practice, into what we do and actually seeing it come to life and what it would look like for each company

(Madison, group interview)

Second, they felt that the academic grounding of the tool lent it a credibility, rigour and ‘weight’, attracting both practitioners and institutional leaders to the tool:

I think from a science centre perspective, the fact that all of this is underpinned by rigorous research has just really helped it become something that they [senior leaders] take on board in a way.

(Barbara, individual interview)

Third, the development and use of the tool through the RPP meant that practitioners were significantly supported over time in their engagement with the tool and in their critical reflections, which, we suggest, will have also contributed to the impact of their reflections on practice.

Practitioners also identified four key challenges to using the tool effectively. The first was limited time to devote to critical reflective practice within a time and resource-pressured professional context (Thompson and Pascal 2012). While the project had provided financial resource to each setting to enable practitioners to have dedicated time to engage, some felt that their wider workload and the global pandemic had restricted their participation (e.g., ‘I haven’t had all the time in the world and this project has been quite squeezed’, Tessa, individual interview).

Second, practitioners found that engaging in critical reflective practice involved discomfort (see also Larrivee 2000). As Barbara confided, critically reflective conversations ‘are difficult at the best of times’ but were especially ‘when everyone’s hypersensitive’ (individual interview). While discomfort is to be expected when reflecting and challenging issues of privilege and power, practitioners found that some colleagues were anxious about ‘being put on the spot’ – that is, they worried that any shortcomings and unintentionally inequitable views or practices might be judged negatively by others. Barbara recounted how, despite being keen to participate, some colleagues had asked not to share examples of their less equitable practice in group collective reflection meetings. As Barbara explained, ‘I’ve been trying to talk to them about it again [to explain] “This is a reflective process. … if we can go through this, it may help us shift in the future”’ (individual interview). As discussed by Mezirow (1997), working with discomfort is a necessary and productive part of critical reflective practice and as Larrivee’s (2000, 304) emphasises, ‘inner struggle is a necessary and important stage of a reflective process’. However, it did require careful management and negotiation by partners to help reassure colleagues and support them through the process.

Third, practitioners working within larger organisations worried that, in the absence of a mainstreamed institutional adoption of critical reflective practice, their progress remained limited and precarious. For instance, Kara worried that in the science centre, the capacity to support and champion critical reflective practice sat largely with Tessa, and was disheartened that their calls for a more mainstreamed approach had not been acted on:

We are saying how important it is what Tessa is doing – but we don’t have anyone shadowing her, working with her. If she goes, it’s gone.

(Kara, group interview).

This concern echoes the recognition in the literature that dominant managerialist culture tends to be antithetical to supporting critical approaches to practice and affording time for reflection (e.g., Issitt 2000). This point was also picked up by Kevin, who recalled how difficult it had previously been to engage zookeepers with equity-focused work. However, he noticed a change over time, as the work undertaken by two members of staff started to translate into changes in zoo practice, leading to wider buy-in:

Once the programmes started to make a real difference to the zoo and what we were looking to achieve, other staff started to buy into the programmes, and it made things much easier operationally to manage

(Kevin, written reflection notes).

Changes to practice seemed to be implemented more easily and holistically in a small setting where a senior leader had been involved in the RPP whereas change was more challenging to mainstream in a large organisation that had no senior leadership membership of the RPP.

Finally, a challenge identified by practitioners from all settings was how their efforts at equitable practice sometimes encountered resistance and push-back from dominant communities and those in power (both within and beyond their organisation). As Scott astutely noted, colleagues who are from privileged communities can feel ‘threatened’ by equity work, and those in senior positions may resist attempts to upset the status quo:

If you, as an individual, are getting involved in equity or programmes that are meant to make the organisation more inclusive or representative, you can feel threatened by it … because it threatens your career. It can threaten your culture and your experience and what you think. We’ve all felt that.

(Scott, group interview)

Often pushback was couched in the notion that equitable practice sits at odds with income generation and financial viability and/or cannot be quantified. For example, Barbara worried how her proposals to support equitable change would face considerable ‘challenge’ if they ‘don’t result in sales’ and Kevin recounted the initial resistance to changes that he and colleagues were trying to introduce until the impact of their work became clearer (‘this largely changed when we started getting stories about how the programmes had affected the youngsters and its purpose became more obvious’, written reflection notes). Cole and Tessa also shared how they had encountered push back and hostility on social media from the public in response to equitable initiatives that they had introduced (such as offering language translations and support for vulnerable groups). Such examples show how critical reflective practice can sit in tension with wider cultural and social power relations (Thompson and Pascal 2012), echoing the point made by Brechin, Brown, and Eby (2000, xi), that critical practice ‘acknowledges that … established voices will often hold sway over newer, alternative ways of seeing things’.

## Discussion and Conclusion

In this paper, we analysed multimodal data from a five-year research-practice partnership project to explore how a new co-designed conceptual tool, *The Equity Compass*, helped support ISL practitioners towards critical reflection and more equitable practice. Using a coding frame derived from a synthesis of the critical reflective practice literature, we explored how the tool helped practitioners to develop and extend their knowledge and understanding and view their practice through a social justice lens that foregrounds intersectional equity issues. We examined how the tool was used by practitioners to support critical reflection in ways that interrogated their own intersectional positionality, questioned previous forms of dominant practice, and supported intentional planning and action towards more participatory approaches that recognised intersectional injustices, valued participant identities and lived experiences and aimed to rework traditional power relations within learning programmes (see Table 2).

### Supporting equitable practice in informal STEM learning

As summarised in Table 2, practitioners developed three new forms of knowledge and understanding of equity and social justice issues, each of which helped feed into the development of three main forms of critical reflection and intentionally equitable practice. These changes were enacted across individual, collective/organisational and field levels, suggesting a breadth and richness of the impact. Practitioners increased their understanding of how intersectional forms of power and privilege produce social inequalities in and beyond ISL and STEM. They used *the Equity Compass* reflective questions to interrogate their own positionality and ask new questions of their own and others’ practice which, in turn, led to advocating for change (e.g., more participatory learning approaches) in their organisation and/or the wider sector. By deepening their understanding of different dimensions of equity, practitioners were better able to critically evaluate and identify the limitations of existing practice and develop more participatory and inclusive forms of practice as a result. The academic grounding and closeness to practice of the tool, plus its use within a supportive RPP context all aided its effective implementation. Yet, practitioners still experienced challenges, such as a lack of time to engage in reflective practice and dealing with discomfort that arises from engaging in equity work. A failure to embed critical reflective practice across the organisation and experiences of resistance from the powerful also threatened their progress and impact.

### The role of the Equity Compass tool

As Rusznyak (2022) discusses in the context of formal education, ‘teachers need powerful and relevant conceptual tools with which to think systematically about their classroom observations and experiences’. Our findings suggest that *the Equity Compass* tool helped support practitioners to move beyond ‘hall of mirrors’ (Fox, Dodman, and Holincheck 2019) forms of reflection and towards forms of critical reflection that helped open up new possibilities for more equitable practice. *The Equity Compass* helped to (1) introduce and familiarise practitioners with new social justice-related concepts and language, supporting their intersectional ‘equity literacy’; (2) foreground unequal power relations and de-centre their own positionality and dominant perspectives, providing a set of reflective questions that supported practitioners to critically re-consider their practice through the lens of equity and (3) provide a practical framework to apply to practice that enabled practitioners to understand and differentiate the equitable potential of different forms of practice in relation to specific dimensions of equity, and to evaluate and map their progress towards more equitable practice.

While the tool helped support practitioners to meaningfully extend their equity-related knowledge, reflection and action across different scales, our paper is also limited in that our data do not follow this impact through to learners and participants within the four spaces. That is, we cannot yet tell what difference the practitioners’ practice might make for young people within their spaces – although this is an area that we hope to explore in the future.

As Rusznyak (2022) usefully argues, the efficacy of tools that aim to prompt ‘deep’ critical reflection is determined by the ways in which participants interpret, negotiate, and enact (or resist) a given tool. Our findings lent support to this argument, showing how the capacity for the Compass to support critical reflection was mediated by interactions between the intersectional identities, experiences and positionality of participants and facilitators – and also by the mode of delivery (in our case, the RPP approach).

### Contributions and implications

In this paper we have sought to augment understanding of how equity-orientated critical reflection and action might be supported in educational settings. Our findings lend empirical support to previous calls regarding the value of critical reflective practice at both individual and collective levels for helping to challenge intersectional injustices in and through educational practice (e.g., Brookfield 1995; Ladson-Billings 1995). Additionally, our synthesised coding frame offers a methodological contribution to help researchers to capture and evidence practitioners’ critical reflective practice.

Our findings lend support to calls for greater investment in critical professional reflection that supports participants to evaluate their practice through the lens of intersectional social justice in order to enhance equitable practice within ISL. While the sector undoubtedly faces intense pressures, as Thompson and Pascal (2012) emphasise, the greater the pressure, the clearer practitioners must be about what we are doing, why and for what purposes. If ISL is to contribute to challenging, rather than reproducing, social injustices, then investment in critical professional reflection among education practitioners is imperative. Such investment could help ISL to unlock its potential to contribute to strategic national goals, such as fostering more inclusive participation in STEM (e.g., UK Government 2021). Our work suggests that tools, such as *the Equity Compass*, when enacted through an RPP approach, can be effective in supporting critical reflection and equitable practice. In particular, we call on UK ISL funders to further support RPPs, which remain relatively rare but offer a valuable way to support more effective, impactful professional practice towards social justice.
